# Genome skimming is a low-cost and robust strategy to assemble complete mitochondrial genomes from ethanol preserved specimens in biodiversity studies

**DOI:** 10.7717/peerj.7543

**Published:** 2019-09-13

**Authors:** Bruna Trevisan, Daniel M.C. Alcantara, Denis Jacob Machado, Fernando P.L. Marques, Daniel J.G. Lahr

**Affiliations:** 1 Department of Zoology, Institute of Biosciences, University of São Paulo, São Paulo, São Paulo, Brazil; 2 Department of Bioinformatics and Genomics / College of Computing and Informatics, University of North Carolina at Charlotte, Charlotte, NC, United States of America

**Keywords:** Mitochondrial genome, Automated assembly, Automated annotation, Poorly preserved samples, High-coverage genome, Diptera, Cestoda

## Abstract

Global loss of biodiversity is an ongoing process that concerns both local and global authorities. Studies of biodiversity mainly involve traditional methods using morphological characters and molecular protocols. However, conventional methods are a time consuming and resource demanding task. The development of high-throughput sequencing (HTS) techniques has reshaped the way we explore biodiversity and opened a path to new questions and novel empirical approaches. With the emergence of HTS, sequencing the complete mitochondrial genome became more accessible, and the number of genome sequences published has increased exponentially during the last decades. Despite the current state of knowledge about the potential of mitogenomics in phylogenetics, this is still a relatively under-explored area for a multitude of taxonomic groups, especially for those without commercial relevance, non-models organisms and with preserved DNA. Here we take the first step to assemble and annotate the genomes from HTS data using a new protocol of genome skimming which will offer an opportunity to extend the field of mitogenomics to under-studied organisms. We extracted genomic DNA from specimens preserved in ethanol. We used Nextera XT DNA to prepare indexed paired-end libraries since it is a powerful tool for working with diverse samples, requiring a low amount of input DNA. We sequenced the samples in two different Illumina platform (MiSeq or NextSeq 550). We trimmed raw reads, filtered and had their quality tested accordingly. We performed the assembly using a baiting and iterative mapping strategy, and the annotated the putative mitochondrion through a semi-automatic procedure. We applied the contiguity index to access the completeness of each new mitogenome. Our results reveal the efficiency of the proposed method to recover the whole mitogenomes of preserved DNA from non-model organisms even if there are gene rearrangement in the specimens. Our findings suggest the potential of combining the adequate platform and library to the genome skimming as an innovative approach, which opens a new range of possibilities of its use to obtain molecular data from organisms with different levels of preservation.

## Introduction

Global loss of biodiversity is an ongoing process that concerns both local and global authorities (Vellend, 2017). Biodiversity loss impacts ecosystem functions, while additionally increasing the knowledge gaps and sampling biases (Asaad et al., 2017; Oliveira et al., 2017). Challenges including habitat loss, overexploitation, climate change, and invasive species are far from a solution (Corlett, 2017; Vellend, 2017). Some advocate the use of natural history collections as a central tool for the study of biodiversity, especially for species that are becoming extinct or increasingly rare (Staats et al., 2013; Kanda et al., 2015). Studies of biodiversity mainly involve traditional methods using morphological characters and molecular protocols predominantly by PCR-based methods. However, conventional techniques are a time consuming and resource demanding tasks (Cameron, 2014a; Yuan et al., 2016). Among molecular approaches, the PCR-based methods are often not successful in recovering genetic data of preserved organisms, due to the fragmented nature of old and poorly-preserved DNA (Heintzman et al., 2014; Timmermans et al., 2016). Also, the lack of genomic resources such as well-established and optimized molecular markers and primers to delimit target amplicons for closely related species may hamper PCR-based methods for non-model organisms (Ekblom & Galindo, 2011; Tilak et al., 2015; Matos-Maraví et al., 2019).

The development of high-throughput sequencing (HTS) techniques has reshaped the way we explore biodiversity and opened a path to new questions and novel empirical approaches (Dodsworth, 2015; Linard et al., 2015; Porter & Hajibabaei, 2018). The use of low-coverage and cost-effective genome-skimming, also known as whole-genome shotgun sequencing (WGS), is one of these techniques. This particular method consists of sequencing the whole genome of an individual at low nuclear genome coverage. The process provides an extensive data set, capable of recovering high-copy fractions of total genomic DNA (organellar genomes, nuclear ribosomal DNA, and other multi-copy elements) through random shearing and inexpensive multiplexing (Berger et al., 2017; Matos-Maraví et al., 2019). The technique is potentially efficient for old museum material and ethanol-preserved specimens (Staats et al., 2013; Maddison & Cooper, 2014; Linard et al., 2016; Grandjean et al., 2017). Despite recent efforts to obtain DNA sequences through HTS protocols from museum specimens (McCormack et al., 2017; Dabney et al., 2013; McCormack, Tsai & Faircloth, 2016) and ethanol material (Raposo do Amaral et al., 2015; Brabec et al., 2015; Brabec et al., 2016; Hartikainen et al., 2016; Vanhove et al., 2018), the potential use of genome skimming for this purpose remains unexplored. There is a variety of suitable HTS sequencing platforms and library options to choose from to use in combination with genome skimming, including PCR-based libraries and PCR-free libraries that are less-error prone but require higher input DNA (≤ one µg) (Twyford & Ness, 2017). Knowing that the preservation level of biological samples can influence the quality of sequencing, it is important to consider the amount of input DNA available. Since researchers often preserve specimens of invertebrates in ethanol, specific protocols are required to obtain high-quality data from low quality or quantity DNA extracts (Tilak et al., 2015; Linard et al., 2016). Therefore, combining the adequate platform and library to the genome skimming technique is an innovative approach, which could overcome most of the limitations highlighted above, opening a new range of possibilities of its use for obtaining molecular data.

The mitogenome has been used as a molecular marker in a great variety of studies (e.g., ecology, evolution, phylogeography and phylogenetics at multiple taxonomic levels; see Avise et al., 1987; Le et al., 2000; Zarowiecki, Huyse & Littlewood, 2007; Avise, 2012; Li et al., 2017). Its popular use throughout those areas could be attributed to its particularity as maternal inheritance, high copy-number, lack of recombination and higher mutation rate when compared to other markers (Ballard & Whitlock, 2004; Hahn, Bachmann & Chevreux, 2013; Yuan et al., 2016; Li et al., 2017). Animal mitochondrial genomes are generally uniform across metazoan groups (Park et al., 2006; Tan et al., 2017): a circular, double-stranded DNA molecule, ranging from 15–20 kb in size, containing circa 37 genes (i.e., two mitochondrially encoded ribosomal RNAs [rDNA], 13 protein-coding genes [PCG] and 22 transfer RNA genes [tRNA]) (Park et al., 2006; Castellana, Vicario & Saccone, 2011; Bernt et al., 2013; Tan et al., 2017). This set of attributes provide to mitogenomes a wide spectrum of informational content, which can be used to answer many biodiversity questions.

Despite the potential of mitogenomes in solving biodiversity questions, the great majority of the studies only targeted a small fraction of this genome. The prevalence of partial sequences of the mitochondrially encoded ribosomal RNAs MT-RNR1 and MT-RNR2 (12S and 16S, respectively), Cytochrome B (MT-CYB) and Cytochrome C Oxidase I (MT-CO1) in many studies can be credited to the existence of “universal primers” that amplified these regions for a whole spectrum of non-model Metazoa taxa. Hence, it is not uncommon to find studies addressing phylogenetic relationships at different levels of divergence—including the interest on detecting cryptic species—based on these markers (Von Nickisch-Rosenegk, Lucius & Loos-Frank, 1999; Zehnder & Mariaux, 1999; Hu et al., 2005; Wickström et al., 2005; Littlewood, Waeschenbach & Nikolov, 2008; Brabec et al., 2016 and Vanhove et al., 2018 to cite a few). However, to date HTS provided the means of sequencing the complete mitochondrial genome in reasonable time and at relative low cost. As a result, the number of genome sequences published has increased exponentially during the last decades (Park et al., 2006; Hahn, Bachmann & Chevreux, 2013; Tan et al., 2017; Raposo do Amaral et al., 2015; Vanhove et al., 2018). With the increase of studies using complete mitogenomes, several authors have recognized the virtues of a greater amount of nucleotide sequence data for inferring robust phylogenies in many taxonomic groups such as mammals (Arnason et al., 2002; Campbell & Lapointe, 2011), birds (Pacheco et al., 2011), insects (Cameron, 2014b), and flatworms (Brabec et al., 2015; Brabec et al., 2016; Maldonado et al., 2017; Vanhove et al., 2018). In addition, we can also relate the power of resolution of the mitogenome to its genome-level characteristics such as gene arrangements and the positions of mobile genetic elements, which are good alternatives to resolve deeper phylogenetic questions (Waeschenbach, Webster & Littlewood, 2012; Guo, 2015; Li et al., 2017).

In spite of its undeniable informational content, whole mitogenomes are still relatively under explored in phylogenetic studies for a multitude of taxonomic groups, especially for those without commercial relevance, non-model organisms, and preserved DNA (Littlewood, Waeschenbach & Nikolov, 2008; Maldonado et al., 2017). The majority of these groups still have poorly understood phylogenetic histories. Two examples of such groups are cestodes in the family Rhinebothriidae and dipterans of the family Streblidae. Rhinebothriideans are exclusively endoparasites of batoid elasmobranchs, while Streblids are highly specialized ectoparasites of bats (Dick, Graciolli & Guerrero, 2016; Ruhnke, Reyda & Marques, 2017). Both groups have intricate historical associations with their hosts; which are of scientific interest of evolutionary biologists engaged in understanding how historical association events shaped the ecology, patterns of association and evolution of these host/parasite systems (Wenzel, Tipton & Kiewlicz, 1966; Brooks, Thorson & Mayes, 1981b; Dick & Patterson, 2006; Tello, Stevens & Dick, 2008; Marques & Caira, 2016).

However, despite the efforts to reconstruct the phylogenies using fragments of mtDNA (or, in the case of rhinebothriideans, only pieces of rRNA genes), their internal relationships remain poorly understood. We could attribute this lack of understanding to a number of factors including the difficulty in extracting DNA from fixed organisms, the low resolution of those markers, and the limited availability of sequenced samples (Dittmar et al., 2006; Petersen et al., 2007; Caira et al., 2014; Ruhnke, Caira & Cox, 2015; Trevisan, Primon & Marques, 2017). Thus, mitogenomics carries the potential to resolve the phylogenetic history in those groups. Here we take the first step to assemble and annotate the genomes from HTS data using a new genome skimming protocol, revealing an opportunity to extend the field of mitogenomics to under-studied organisms.

## Methods

### Taxon sampling

We fixed all samples in 96% ethanol and stored them at –20 °C until we performed the genomic DNA extractions. Hologenophores (sensu Pleijel et al., 2008) from Rhinebothriidae were deposited at MZUSP (Museu de Zoologia da Universidade de São Paulo, Universidade de São Paulo, São Paulo, SP, Brazil). We collected the batflies in Brazil, *Paradyschiria parvula* Falcoz, 1931 from Base de Estudos do Pantanal, Passo do Lontra, Corumbá, Mato Grosso do Sul (19°34′48.0″S, 57°01′15.1″W) in 2013 and *Paratrichobius longicrus* (Miranda Ribeiro, 1907) from Núcleo Pedra Grande, Parque Estadual Cantareira, São Paulo, São Paulo (23°26′10.9″S, 46°38′07.8″W) in 2017. We collected the hosts following the permit guidelines issued by Sistema de Autorização e Informação em Biodiversidade - SISBIO (5184-1, issued in 2013, Brazil to Gustavo Graciolli from Universidade Federal do Mato grosso do Sul to sample *Paradyschiria parvula* and by SISBIO and by Secretaria do meio Ambiente - SMA to sample *Paratrichobius longicrus* (55242-1 and 260108–008.107/2016, respectively) both issued in 2016, Brazil to Daniel Máximo Corrêa de Alcantara. Under those permits, we captured these hosts using mist nets, opened at ground level in trails and other locations near to bodies of water during 6 h after sunset, and checked them every 30 min. We examined the bats for ectoparasites manually or with the aid of tweezers. Bat collection procedures were approved by Comissão de Ética no Uso de Animais, Instituto de Biociências, USP (Proc. 16.1.448.41.6).

We obtained *Anindobothrium anacolum* Marques, Brooks & Lasso, 2001 (Voucher MZUSP 7968) and *Rhinebothrium reydai* Trevisan & Marques, 2017 (Voucher MZUSP 7969) from the spiral intestines of the stingray *Styracura schmardae* from Trinidad & Tobago (Maracas, San Juan-Laventille, 10°45′N, 61°26′W) in 2014 and from Panama (Almirante, Bocas del Toro, 9°17′N, 82°20′W) in 2015, respectively. We collected these hosts using spears following the permit guidelines issued by the Ministry of Food Production—Fisheries Division (issued in September 2014, Trinidad & Tobago to F.P.L. Marques) and by the Autoridad Nacional del Ambiente—ANAM (SE/A-101-14, issued in December 2014, Panama to F.P.L. Marques), respectively. Further details on the collection of hosts and specimens preparation is available in Trevisan, Primon & Marques (2017) and Marques & Reyda (2015).

### DNA extraction

For Streblidae, we extracted DNA using the Qiagen DNeasy Blood & Tissue Kit (Qiagen). Since it is common the abdomen of Streblidae contain the host’s blood, we separated the thorax of each specimen from the abdomen, and only the thorax with the head and legs were used to avoid contamination. The DNA was eluted in 200 µl of a buffer solution, repeating the elution step twice with the addition of 100 µl each time for increased DNA yield. After extraction, we stored the thorax with the head and legs in ethanol, together with the specimen abdomen, and cataloged as specimen vouchers.

For Rhinebothriidae, we extracted the DNA from the middle portion of the strobila of each specimen, which was removed and allowed to air dry for about 5 min at room temperature. We extracted total genomic DNA using Agencourt DNAdvance—Nucleic Acid Isolation Kit (Beckman Coulter, Brea, CA, USA) following the manufacturer’s instructions. We prepared scolices and posterior portions of strobila from specimens used in molecular analyses as whole mounts following traditional protocols (Trevisan, Primon & Marques, 2017).

We employed standard precautions to minimize contamination throughout, such as using exclusive pipettes with filter tips and bleaching all the instruments used in DNA extraction. We measured the purity and amount of DNA extractions using a NanoDrop 2000 spectrophotometer (Thermo Fisher Scientific, Waltham, MA, USA) and Qubit 2.0 Fluorometer using Qubit high sensitivity dsDNA assays (Life Technologies, Carlsbad, CA, USA).

### Library preparation and sequencing

We used Nextera XT DNA Library Preparation Kit (Illumina) to prepare indexed paired-end (PE) libraries according to the manufacturer’s protocol. We chose Nextera XT to prepare the libraries because the fabricator optimized the protocol for one ng (5 × 0.2 ng/µl) of input DNA in total. The low amount of input DNA provides a powerful tool for working with a variety of samples that yield either small or copious amounts of tissue. This library is also likely to be suitable for DNA extractions from samples of model and non-model taxa with different ages of fixation, especially for small genomes (≤5 Mb), PCR amplicons, and plasmids.

Before starting libraries preparation, we diluted DNA extracts in Milli-Q water to 0.2 ng/µl, after which we checked the concentration in a Qubit 2.0 Fluorometer. We used a low-cost method to determine the quality and size of the sequencing libraries, as performed in Kang et al. (2017). The technique consists of PCR amplification of the library, using Illumina adapter primers, checking amplicons for quality and size by standard agarose gel electrophoresis. We prepared a PCR master mix with the appropriate volume for each sample, containing 5 µl of KAPA Taq ReadyMix PCR Kit, 3 µl of Milli-Q water, 0.5 µl of Illumina forward primer (10 µM), 0.5 µl of Illumina reverse primer (10 µM) and 1 µl of DNA template. Then, we run PCR using the following protocol: 45 s of initial denaturation at 98 °C; 20 cycles of 25 s of denaturation at 98 °C, 30 s of annealing at 47 °C, and 1 min and 30 s of extension at 72 °C; 3 min of final extension at 72 °C; and hold at 4 °C. Subsequently, we examined these PCR products in 1.8x TBE agarose gel electrophoresis. We determined the sequencing library concentrations on Qubit 2.0 Fluorometer using Qubit high sensitivity dsDNA. Library normalization was done manually, diluting libraries to the same concentration (four nM) before volumetric pooling.

We sequenced the samples of Rhinebothriidae and Streblidae in two different Illumina platforms. Samples of Streblidae were sequenced alone using an Illumina MiSeq System, with a Reagent Kit v3 to generate PE reads of 300 bp. Since it is possible to sequence 24–30 million reads with the specifications used to run the Illumina MiSeq System, we pooled up to two DNA libraries. The samples of Rhinebothriidae were sequenced using an Illumina NextSeq 550 System, with a High-Output Kit to generate PE reads of 150 bp. This system can sequence up to 800 million reads with these specifications, allowing us to pool up to 35–40 DNA libraries. Thereby, each lane of the Illumina NextSeq 550 also received 33 additional libraries. We based the calculation of the number of reads required per sample to recover the complete mitochondrial genome on Richter et al. (2015). We performed all DNA sequencing in the Core Facility for Scientific Research—University of São Paulo (USP) (CEFAP-USP).

### Computational resources

We executed *in silico* procedures using “ACE”, an SGI rackable computer cluster housed in the Museum of Zoology of the University of São Paulo. Select servers had four 2.3 GHz Operon CPUs with 16 cores each and 256 or 516 GB of memory. The software environment in ACE consists of a SUSE Linux Enterprise Server with SGI Performance Suite, SGI Management Center and PBS Pro Job Scheduler. We were able to reconstruct each genome using a single core and ca. 20 GB of memory.

### Quality control and mitogenome assembly

We pre-processed the raw reads from each pair using a series of UNIX commands. We trimmed and filtered the sequences using the HTQC toolkit (Yang et al., 2013) a home-made Python script (selectTiles.py, see Machado, Lyra & Grant 2016) that automates tiles selection. We evaluated the quality of filtered reads with FASTQC (Andrew, 2010). The assembly protocol received only filtered PE reads. We described the complete quality control protocol below and the step-by-step procedures are given in Machado, Lyra & Grant (2016, Appendix S1).

We performed the sequence assembly using a baiting and iterative mapping strategy based on MIRA v4.0 Chevreux, Wetter & Suhai (1999) and a modified version of MITOBIM.PL v1.6 (Hahn, Bachmann & Chevreux, 2013), following the guidelines described in Machado, Lyra & Grant (2016, Appendix S2). We applied the same search parameters to every assembly but used different baits depending on the class of the specimen. The reference mitogenome sequence of the house fly (*Musca domestica* L., GenBank Accession Number KM200723) was the bait for all streblids, and the reference mitogenome sequence of the beef tapeworm (*Taenia saginata* Goeze, 1782, GenBank Accession Number NC_009938) was the bait for the assembly of cestodes. Finally, we inferred the completeness of each new putative mitogenome (i.e., sequence circularization) using the AWA program and the contiguity index statistics described in Machado et al., 2018; the AWA beta version is available at https://gitlab.com/MachadoDJ/awa.

We mapped the raw sequence reads back to the putative mitogenome selected by AWA with Bowtie2 v2.2.6 (Langmead & Salzberg, 2012) using the local alignment algorithm and the highest sensitivity setting. We set the threshold for base calling on the consensus sequence to bases that match at least 99% of the sequences, with a minimum coverage per position of three sequences.

### Mitogenome annotation

We parsed the assemblies in CAF format using a home-made Python script (parseCaf.py; see Machado, Lyra & Grant, 2016) to extract DNA data and evaluate the coverage and quality of each mtDNA element. Preliminary *de novo* mitogenome annotation used the mitochondrial genome annotation server MITOS2 (Bernt et al., 2013, available at http://mitos2.bioinf.uni-leipzig.de), changing the genetic code accordingly (transl_table=9 for flatworm, transl_table=4 for insects).

We applied three different strategies independently to corroborate the annotations of coding genes. We used the BLAST (Altschul et al., 1990) to search a selected database of mitochondrial peptides from UniProt/Swiss-Prot (The UniProt Consortium, 2016; UniProt resources are available at https://www.uniprot.org/). We executed a second comparison between reference amino acid sequences and the new nucleotide sequences with GeneWise (Birney, Clamp & Durbin, 2004). Finally, we also applied TransDecoder (see Hahn, Bachmann & Chevreux, 2013; the program is available at https://github.com/TransDecoder) to identify candidate coding regions and compare the outputs from these programs to propose the final annotations.

We performed additional search and validation of tRNA sequences using ARWEN (Laslett & Canbäck, 2007) and tRNAscan-SE (Lowe & Eddy, 1997; Schattner, Brooks & Lowe, 2005). We confirmed and edited manually the automated annotation by comparison to published reference mitogenomes of flies and tapeworms. We annotated the control region (CR) with sequence similarity searches in BLAST using default parameters.

**Figure 1:**
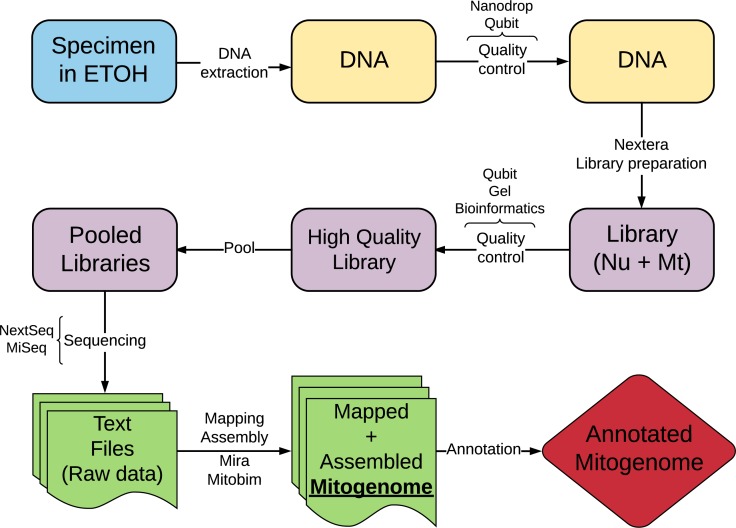
Schematic workflow of the new protocol proposed in this study.

Annotation of cestode mitogenomes was less straightforward compared to streblid organelles and required a more complex strategy. This was needed because some software mentioned above and used for annotation have not yet fully implemented the traditional codon table for flatworms (transl_table=9). Besides, nucleotide and amino acid reference sequences were not available. These limitations reduced the efficiency of the annotations of flatworm sequences, resulting in some wrong start or end positions or missing genes. Although they did not impede our semi-automatic annotation strategy, the procedure was very time-consuming and required manual curation. Figure 1 shows a summary of the workflow of the protocol we proposed in this study.

## Results

### DNA extraction, library preparation and genome sequence

We obtained sufficient DNA quantities for all samples (Table 1), but we required some additional protocols to achieve the initial concentration for library preparation. We diluted the samples from *A. anacolum* and *R. reydai* because they contained a DNA concentration higher than initially required. The DNA concentration obtained for *P. longicrus* was closer to the amount necessary to prepare the library, and therefore its DNA was not diluted. In the case of *Paradyschiria parvula*, the DNA concentration was too low (≤0.50 ng/ml). In this particular case, we concentrated the extracted DNA using Agencourt AMPure XP and eluting the DNA in 15 µl. This protocol allowed us to reach the concentration close to that necessary to start the library preparation, without the need for dilution (Table 1). The sequence on Illumina MiSeq resulted in a total of 13.98 mi PE reads for *P. parvula* and 16.82 mi PE reads for *P. longicrus*. For Rhinebothriidae, the sequence on Illumina NextSeq resulted in a total of 11.81 mi PE reads for *A. anacolum* and 7.04 mi PE reads for *R. reydai*.

**Table 1:** Concentration of DNA in the extraction and library preparation from the specimens included in this study. The measurements were performed in Qubit 2.0 Fluorometer and the values are given in ng/µl. A, after DNA extraction; B, after using Agencourt AMPure XP; Dilution, after diluting the DNA extracted to start the library preparation; Final, after library preparation with Nextera XT.

**Species**	**Extraction**		**Library Prep**	
	**A**	**B**	**Dilution**	**Final**
*Paradyschiria parvula*	≤0.1	0.184	–	2.12
*Paratrichobius longicrus*	0.354	–	–	3.34
*Anindobothrium anacolum*	18.6	–	0.208	14.2
*Rhinebothrium reydai*	9.08	–	0.260	12.9

### Mitogenome assembly

**Figure 2:**
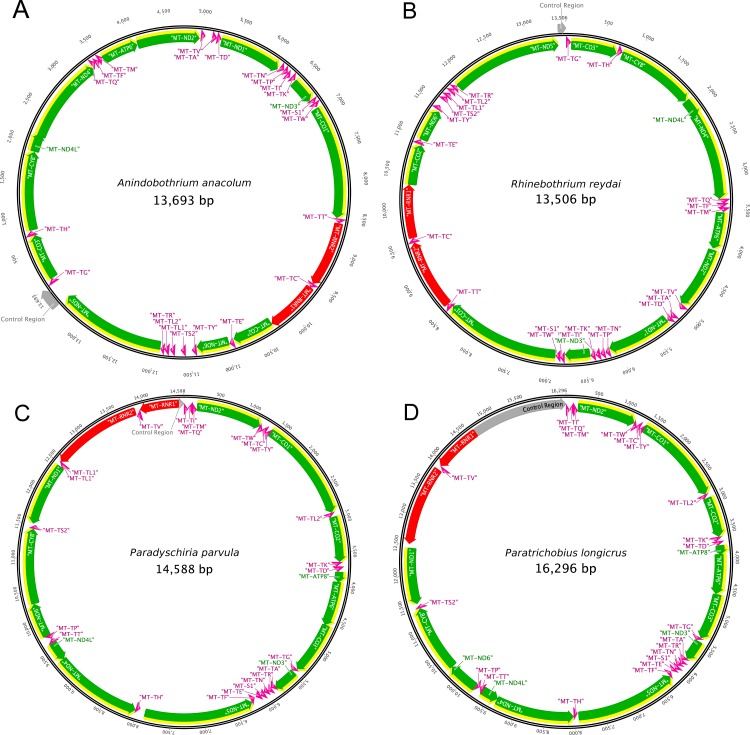
Graphical representation of the mitogenomes of (A) *Anindobothrium anacolum*, (B) *Rhinebothrium reydai*, (C) *Paradyschiria parvula*, and (D) *Paratrichobius longicrus*. Grey: control region; yellow: CDS; green: genes; red: rRNA; pink: tRNA.

The present study is the first to report the complete mitochondrial genome of species from the two families, Streblidae and Rhinebothriidae. Figure 2 illustrates the annotation and gene map of the new mitogenomes. The complete mitogenome sequences obtained for the two species of Streblidae were 14,588 bp and 16,296 bp for *Paradyschiria parvula* (GenBank accession no. MK896865) and *Paratrichobius longicrus* (GenBank accession no. MK896866), respectively. The average coverage for the assemblies of Streblidae was high, 1,749.6 for *Paradyschiria parvula* and 6,355 for *Paratrichobius longicrus*. Their mitogenome length conforms with those found in other Diptera, typically 14–19 kb (Li et al., 2015). The total number of mapped sequences for *Paradyschiria parvula* was 96,404 whereas *Paratrichobius longicrus* shown a higher value of 695,060. However, circularity tests of the mitogenome of *Paradyschiria parvula* presented a higher average coverage, contiguity, and score than *Paratrichobius longicrus* (329 vs. 37.3, 322.3 vs. 36.3 and 1.43 vs. 6.79, respectively) (Table 2). The lower score of *Paratrichobius longicrus* is bound to be affected by the ambiguous nucleotides obtained from our conservative approach to base calling (detailed below).

The mitogenome sequences obtained for specimens of Rhinebothriidae, *A. anacolum* (GenBank accession no. MK887326) and *R. reydai* (GenBank accession no. MK896864), were 13,693 bp and 13,506 bp in length with 743.4 and 258.5 coverage, respectively. Their mitogenome size follows the pattern observed previously for tapeworms (13–15 kb) (Li et al., 2017). The total number of mapped sequences for *A. anacolum* was 83,969 and *R. reydai* shown a smaller value of 28,583. We observed the opposite for the contiguity index in which *A. anacolum* had a lower value in comparison to *R. reydai* (153.2 vs. 313.8, respectively). The GC content and quality of the sequences was similar for both species (30.4% vs. 35.8% and 34.0 vs. 32.4, respectively). Overall the scores *A. anacolum* and *R. reydai* were close to 0, which should be considered as another good indicator of quality (non-ambiguity) (Table 2).

**Table 2:** General assemble statistics. Q1 and Q3 indicate 1st and 3rd quartiles for coverage, respectively. Asterisks indicate indexes calculated during circularization tests in AWA (Machado et al., 2018) using the 50 nucleotides flanking each end of the putative mitochondrion contig.

					AWA			
Species	**Length**	**GC%**	No. of sequences	Avg. Coverage **(Q1 : Q3)**	Avg. Coverage*	Avg. Contiguity*	**Avg.** Quality*	Bowtie2 Score*
*P. parvula*	14,588	21.4	96,404	1,749.6	329	322.3	35.8	−1.43
				(841 : 2,133)				
*P. longicrus*	16,296	17.9	695,060	6,355.0	37.3	36.3	36.0	−6.79
				(5,506 : 7,945)				
*A. anacolum*	13,693	30.4	83,969	743.4	157.3	153.2	34.0	−0.92
				(526 : 962)				
*R. reydai*	13,506	35.8	28,583	258.5	318.9	313.8	32.4	−2.45
				(216 : 305.3)				

### Mitogenome organization and structure

#### Streblidae

The mitogenome of both species contains 37 genes, including 13 PCGs, 22 tRNA genes, two rRNA genes and the CR. We recovered 71 bp of the CR for *P. parvula* and 1,579 bp for *P. longicrus*. For both species, 23 genes are encoded on the majority strand (14 tRNAs and nine PCGs), while the minority strand encodes the remaining 14 genes (eight tRNAs, the two ribosomal RNAs, and four PCGs) (Fig. 2). We must note a bias in the nucleotide composition of the mitogenome toward A and T, with a GC content of 21.4% for *P. parvula* and 17.9% for *P. longicrus* (Table 2). Except for MT-CO1, each of the thirteen PCGs had the canonical start codon ATT (encoding Ile) or ATG (encoding Met). We identified the MT-CO1 start codon as TCG (encoding Ser). We found incomplete stop codons (T) only in MT-ND4 for *P. parvula*. All other PCGs have the complete stop codons TAA or TAG in both species, being TAG found only for MT-CYB. We also found several stop codons TGA within all PCGs.

#### Rhinebothriidae

Each mitogenome contains 36 genes, including 12 protein-coding genes (MT-ATP6, MT-CO1-3, MT-CYB, MT-ND1-6 and MT-ND4L), 22 transfer RNA genes (tRNA), two ribosomal RNA genes (RNR1-2) and one CR. As previously reported for other Neodermata mitogenomes, the specimens from Rhinebothriidae also lacks the ATP8 gene, which is found in other metazoan mitogenomes (Le, Blair & McManus, 2002; Guo, 2016; Zhao et al., 2016; Li et al., 2017; Egger, Bachmann & Fromm, 2017). We noticed that the nucleotide composition of the mitogenome is biased towards A and T, with a GC content of 35.8% for *R. reydai* and 30.4% for *A. anacolum* (Table 2). All genes are encoded in the same strand and transcribe from the same direction. The gene order in *A. anacolum* and *R. reydai* follows the typical organization of cestodes, except by the rearrangements of some tRNA genes and by the number of non-coding regions (NCR) (i.e., one). Despite the slightly differences, these results reinforces previous evidences that cestode gene order is extremely conserved (Nakao, Sako & Ito, 2003; Li et al., 2017; Zhang et al., 2017). The reader should note that annotations described here for the mitogenomes of flatworms derive from a semi-automatic and manually curated annotation protocol but, given the specific challenges involved in annotating this mitogenomes, some of the authors from this publication prepared a pipeline dedicated to their annotation, which is available at https://gitlab.com/MachadoDJ/cma.

## Discussion

### Coverage variation

The overall quality and alignment score of the assembly of the four mitogenomes indicate that even with relatively lower coverage values, it is possible to recover the mitochondrial genome with this protocol. Given our conservative approach towards base calling, we left some ambiguous nucleotides in six tRNAs, two rRNAs, 11 PCGs and the CR of the mitogenome of *P. longicrus*. However, the number of these ambiguities is within our expectations given the procedures described here and the expected variation in the percentage of mitochondrial among different libraries.

As expected, there was a wide variation on the number of reads mapped to each mitochondrial genome. The ratio between the number of mitochondria and the genome size of the organism influences the number of mitochondrial reads in a library (Richter et al., 2015). In tunicates, for instance, a lower proportion of mitochondria to nuclei in tissues is correlated to reduced numbers of mitochondrial reads (Tilak et al., 2015). The mitogenome coverage may even correlate negatively with the amount of DNA used for sequencing, since higher amounts of DNA can increase the chance of introducing more nuclear reads into the library, reducing the number of mitochondrial reads. Furthermore, the number of multiplexed species per sequencing can also influence the number of mitochondrial reads sequenced and the average coverage (Richter et al., 2015; Tilak et al., 2015). Therefore, the number of recovered mitochondrial reads in different genome-skimming experiments can vary.

The quality of the assembly around the 50 bp flanking each end of the putative mitogenomes is also presumed to vary. For the assembly of *P. longicrus* mitogenome, which has the lowest coverage and contiguity score in AWA, our analysis found *k*-mer lengths that obtained an average coverage higher than that presented in Table 2 (up to 207.73x). However, the identity and quality of these alignments were much lower, and therefore they were not selected by AWA software based on the combined criterion of read coverage, read contiguity, average quality, and alignment scores.

### Streblidae mitogenome order rearrangements

Compared to other dipterans, the gene order and sizes follows the typical organization reported for this group (Nelson et al., 2012; Cameron, 2014b; Li et al., 2015; Pu et al., 2017). Among flies, ATG (Met) and ATT (Ile) are frequently found as start codons (Li et al., 2015), which is congruent with our results. On the other hand, start codons for MT-CO1 are usually considered non-canonical in holometabolans, though TCG is widely reported for MT-CO1 in Diptera (Cameron et al., 2007; Nelson et al., 2012). The stop codon most commonly found in Diptera is TAA. However, partial stop codon T has been reported in many insect mitogenomes and is completed to a full TAA stop codon via post-transcriptional polyadenylation (Li et al., 2015). Moreover, some authors have already reported that translation termination might reassign the codon UGA to code for tryptophan or cysteine (Alkalaeva & Mikhailova, 2017), which may be the case in this study. Even though the CR can be variable in both size and nucleotide composition, the difference in size between the two species of Streblidae is striking. More study are important to investigate such difference, since cases of duplication in the CR are known in different groups, including insects (Cameron, 2014b; Yan et al., 2012).

Although Diptera is one of the most extensively sequenced orders within Insecta, the number of complete mitogenomes is low given its diversity (Liu et al., 2017; Narayanan Kutty et al., 2019). Streblidae belongs to the Calyptratae clade, one of the most species-rich group within Diptera with over 22,000 species. Calyptratae is divided into three superfamilies: Hippoboscoidea (to which Streblidae belongs), Muscoidea and Oestroidea (Narayanan Kutty et al., 2019). Currently, 231 mitochondrial genome sequences of Diptera are included in the NCBI GenBank Organelle Genome Resources (see also Benson et al., 2008). However, 51 of these mitochondrial genomes are from Calyptrates, while 41 are from Oestroidea, nine from Muscoidea and only one from Hippoboscoidea. It is clear that the Calyptratae clade is poorly represented, and the two new sequences will allow a significant gain not only for the family in question but also at many levels in insect systematics.

### Cestode mitogenome order rearrangements

The most recent and comprehensive study on the diversity of mitogenomes in cestodes was published by Li et al. (2017). In this study, the authors included 54 mitogenomes representing 5 of the 18 orders presently recognized for cestodes (following Caira et al., 2014). The study included members of Caryophyllidea, Diphyllobothriidea, Bothriocephalidea, Onchoproteocephalidea and Cyclophyllidea; however, the latter order comprised 75% (41) of the species included in the study. Hence, this is the first attempt to document the diversity of mitogenomes within this group.

The authors considered that mitogenome gene order is extremely conserved in cestodes. Their assertion was based on the observation that all mitogenomes studied could be attributed to 4 categories based on the arrangements coding regions and tRNAs. According to Li et al. (2017), caryophyllideans possesses a Category I mitogenome from which Category II derived by a transposition event in which the region *tRNA*^*Leu*(*CUN*)^ − *tRNA*^*Ser*(*UCN*)^ − *tRNA*^*Leu*(*UUR*)^ translocated to the 3′ end of the four genes *cox*2 − *tRNA*^*Glu*^ − *Nad*6 − *tRNA*^*Tyr*^. Following, two other categories would have derived from Category II; one by a tandem duplication and random loss (TDRL) event that generated the region *tRNA*^*Leu*(*CUN*)^ − *tRNA*^*Leu*(*UUR*)^ − *tRNA*^*Tyr*^ − *tRNA*^*Ser*(*UCN*)^—Category III; and the other by a simple transposition event generating the region *tRNA*^*Ser*(*UCN*)^ − *tRNA*^*Leu*(*CUN*)^—Category IV.

Although Li et al.’s (2017) study should be considered a preliminary account of the diversity of mitogenomes within cestodes, there appears to be some phylogenetic congruence among the patterns of gene rearrangements that can generate some predictions for future studies. The transposition that characterizes their Category II was found in all polyzooic orders included in the study (i.e., Diphyllobothriidea, Bothriocephalidea, Onchoproteocephalidea and Cyclophyllidea). The hypothesized TDRL resulting in the region *tRNA*^*Leu*(*CUN*)^ − *tRNA*^*Leu*(*UUR*)^ − *tRNA*^*Tyr*^ − *tRNA*^*Ser*(*UCN*)^ found in three species of *Schyzocotyle* could be a putative synapomorphy for the genus, if not for the order Bothriocephalidea. Finally, the transposition leading to the region *tRNA*^*Ser*(*UCN*)^ − *tRNA*^*Leu*(*CUN*)^ could be a synapomorphy for acetabutate cestodes (sensu Caira et al., 2014)—although it seems to be reverted to the ancestral state (*tRNA*^*Leu*(*CUN*)^ − *tRNA*^*Ser*(*UCN*)^) in taeniids.

Our results confirm some of these predictions. Rhinebothriideans are considered to have derived after bothriocephalideans and the order is considered to be sister to a large clade comprised by six orders including Onchoproteocephalidea and Cyclophyllidea (see Caira et al., 2014). As predicted the mitogenomes of *Anindobothrium anacolum* and *Rhinebothrium reydai* share the region *tRNA*^*Ser*(*UCN*)^ − *tRNA*^*Leu*(*CUN*)^ found in acetabutate cestodes, with the exception of teaniids (i.e., Category IV). However, contrary to most mitogenomes known to date for cestodes, our results indicate that rhinebothriideans have only one non-coding regions (NCRs), an attribute also found in the taeniid *Hydatigera taeniformis* (Batsch, 1786) (Li et al., 2017). However, taeniids seem to have reverted to the ancestral gene arrangement of Category II.

Li et al. (2017) acknowledged the possibility that the diversity of arrangements is underestimated given the taxonomic representativity of their dataset. In fact, when NCRs are taken into account, we found that there are few arrangements that were not considered by the authors. For instance, the bothriocephalid *Schyzocotyle acheilognathi* posses a third NCR not found in any other cestode. Within Category IV, there are at least seven distinct arrangements if you consider the position of NCRs; *A. anacolum* and *R. reydai* yet have a different one. We predict that as we compile mitogenomes for cestodes, we will have a better understanding of the rearrangement events associated with the diversity of the group. To achieve this goal, it would be desirable to have representatives of all major lineages of cestodes and access variability in different taxonomic levels. We also think that special attention should be given to homology statements of NCRs since we already have an indication that the number of NCRs differ within the groups (see Li et al., 2017). Finally, we think that once we achieve the goals above and contextualize mitogenome rearrangements within a phylogenetic context we might uncover new synapomorphies for cestode taxa.

### Applicability of the method

Our results illustrated that the proposed protocol can successfully assemble mitochondrial sequences from genome skimming raw data of non-model organisms. We assembled the whole mitogenomes even if there were gene rearrangements, which is reinforced by the contiguity index supporting the circularization of those mitochondria.

The main advantage of this protocol is the possibility to start from a low concentration of DNA extracts (Table 1), circumventing the need for prior enrichment and can work well on samples with different levels of preservation. We believe that the critical point of this advantage lies in the library preparation kit used.

Many studies with genome skimming have used methods in which the user shears the genomic DNA through ultrasonication (Kocher et al., 2014; Richter et al., 2015) or the library prep kit requires a DNA input ≥50 ng (Kocher et al., 2016; Linard et al., 2015). Such methods would probably make it impossible to sequencing some of the samples used in this study (Table 1). For mitochondrial sequencing, Nextera XT is commonly used in conjunction with enrichment methods *via* organelle isolation (Grandjean et al., 2017) or PCR amplification (Li et al., 2015; Foster et al., 2017), but not with genome skimming techniques. Although the Nextera XT is designed for small genomes (≤5 Mb), such as genomes of bacteria and viruses, it has been used to recover mostly plastomes from plants (Burke et al., 2016).

Based on our results, we expect that the methods described here will be valuable to researcher aiming towards sequencing metazoan mitogenomes. The workflow is time-saving, and it is possible to go from DNA to the pool library in a single day. Moreover, researchers can certainly apply this protocol to other non-models organisms, in addition to old historical specimens or specimens that usually generate low concentrations of DNA from the extractions. We have demonstrated that mitochondrial genomes can be generated efficiently from different sequencing strategies, using Illumina MiSeq (two samples and PE reads of 300 bp) and Illumina NextSeq (35 samples and PE reads of 150 bp). Thus, the user can adjust the procedure costs by designing a multiplex pooling strategy that sequences the desired number of samples with suitable coverage.

## Conclusion

The proposed method is an excellent solution to obtain low cost/large scale molecular data in biodiversity studies. Combining the adequate platform and library to the genome skimming is an innovative approach, opening a new range of possibilities of its use in obtaining molecular data from organisms with different levels of preservation. The principal advantages from our approach are: (i) it requires low amount of input DNA (0.2 ng/µl), which allows the use of organisms with preserved DNA; (ii) it does not depends on specific primers and is not affected by gene rearrangement; and (iii) it is time-saving and cost-effective, leading to high-quality complete sequence assemblies.

##  Supplemental Information

10.7717/peerj.7543/supp-1 Supplemental Information 1 Assembled mitogenomes deposited in GenBank This file is for review only, these are the assembled mitogenomes that are being deposited in genbank. They are not available in GenBank yet due to the embargo.

## Additional Information and Declarations

### Field Study Permissions

The following information was supplied relating to field study approvals (i.e., approving body and any reference numbers): Bat hosts were collected following the permit guidelines issued by Sistema de Autorização e Informação em Biodiversidade - SISBIO (5184-1) and by Secretaria do meio Ambiente (SMA 55242-1 and 260108–008.107/2016).

### DNA Deposition

The following information was supplied regarding the deposition of DNA sequences: DNA sequences are available at GenBank: MK887326, MK896864 –MK896866, at BioProject using ID PRJNA540940, and at BioSample via accessions SAMN11490545 –SAMN11490548.

### Data Availability

The following information was supplied regarding data availability: Data is available at Genbank under SRA numbers: SRR9006449, SRR9006450, SRR9006453, SRR9006454, SRR9006455, SRR9006456, SRR9006458, SRR9006459.

## References

[ref-1] Alkalaeva E, Mikhailova T (2017). Reassigning stop codons via translation termination: how a few eukaryotes broke the dogma. BioEssays.

[ref-2] Altschul SF, Gish W, Miller W, Myers EW, Lipman DJ (1990). Basic local alignment search tool. Journal of Molecular Biology.

[ref-3] Andrew S (2010). http://www.bioinformatics.babraham.ac.uk/projects/fastqc.

[ref-4] Arnason U, Adegoke JA, Bodin K, Born EW, Esa YB, Gullberg A, Nilsson M, Short RV, Xu X, Janke A (2002). Mammalian mitogenomic relationships and the root of the eutherian tree. Proceedings of the National Academy of Sciences of the United States of America.

[ref-5] Asaad I, Lundquist CJ, Erdmann MV, Costello MJ (2017). Ecological criteria to identify areas for biodiversity conservation. Biological Conservation.

[ref-6] Avise JC (2012). Molecular markers, natural history and evolution.

[ref-7] Avise JC, Arnold J, Ball RM, Bermingham E, Lamb T, Neigel JE, Reeb CA, Saunders NC (1987). Intraspecific phylogeography: the mitochondrial DNA bridge between population genetics and systematics. Annual Review of Ecology and Systematics.

[ref-8] Ballard JWO, Whitlock MC (2004). The incomplete natural history of mitochondria. Molecular Ecology.

[ref-9] Benson DA, Karsch-Mizrachi I, Lipman DJ, Ostell J, Sayers EW (2008). GenBank. Nucleic Acids Research.

[ref-10] Berger BA, Han J, Sessa EB, Gardner AG, Shepherd KA, Ricigliano VA, Jabaily RS, Howarth DG (2017). The unexpected depths of genome-skimming data: a case study examining Goodeniaceae floral symmetry genes. Applications in Plant Sciences.

[ref-11] Bernt M, Donath A, Jühling F, Externbrink F, Florentz C, Fritzsch G, Pütz J, Middendorf M, Stadler PF (2013). MITOS: improved de novo metazoan mitochondrial genome annotation. Molecular Phylogenetics and Evolution.

[ref-12] Birney E, Clamp M, Durbin R (2004). GeneWise and genomewise. Genome Research.

[ref-13] Brabec J, Kostadinova A, Scholz T, Littlewood DTJ (2015). Complete mitochondrial genomes and nuclear ribosomal RNA operons of two species of Diplostomum (Platyhelminthes: Trematoda): a molecular resource for taxonomy and molecular epidemiology of important fish pathogens. Parasites & Vectors.

[ref-14] Brabec J, Kuchta R, Scholz T, Littlewood DTJ (2016). Paralogues of nuclear ribosomal genes conceal phylogenetic signals within the invasive Asian fish tapeworm lineage: evidence from next generation sequencing data. International Journal for Parasitology.

[ref-15] Brooks DR, Thorson TB, Mayes MA (1981b). Freshwater stingrays (Potamotrygonidae) and their helminth parasites: testing hypothesis of evolution and coevolution. Advances in cladistics, proceedings of the first meeting of the Willi Hennig Society.

[ref-16] Burke SV, Wysocki WP, Zuloaga FO, Craine JM, Pires JC, Edger PP, Mayfield-Jones D, Clark LG, Kelchner SA, Duvall MR (2016). Evolutionary relationships in panicoid grasses based on plastome phylogenomics (Panicoideae; Poaceae). BMC Plant Biology.

[ref-17] Caira JN, Jensen K, Waeschenbach A, Littlewood D (2014). An enigmatic new tapeworm, *Litobothrium aenigmaticum* sp. nov.(Platyhelminthes: Cestoda: Litobothriidea), from the pelagic thresher shark with comments on development of known *Litobothrium* species. Invertebrate Systematics.

[ref-18] Cameron SL (2014b). Insect mitochondrial genomics: implications for evolution and phylogeny. Annual Review of Entomology.

[ref-19] Cameron SL (2014a). How to sequence and annotate insect mitochondrial genomes for systematic and comparative genomics research. Systematic Entomology.

[ref-20] Cameron SL, Lambkin CL, Barker SC, Whiting MF (2007). A mitochondrial genome phylogeny of Diptera: whole genome sequence data accurately resolve relationships over broad timescales with high precision. Systematic Entomology.

[ref-21] Campbell V, Lapointe F-J (2011). Retrieving a mitogenomic mammal tree using composite taxa. Molecular Phylogenetics and Evolution.

[ref-22] Castellana S, Vicario S, Saccone C (2011). Evolutionary patterns of the mitochondrial genome in Metazoa: exploring the role of mutation and selection in mitochondrial protein–coding genes. Genome Biology and Evolution.

[ref-23] Chevreux B, Wetter T, Suhai S (1999). Genome sequence assembly using trace signals and additional sequence information.

[ref-24] Corlett RT (2017). A bigger toolbox: biotechnology in biodiversity conservation. Trends in Biotechnology.

[ref-25] Dabney J, Knapp M, Glocke I, Gansauge M-T, Weihmann A, Nickel B, Valdiosera C, García N, Pääbo S, Arsuaga J-L, Matthias M (2013). Complete mitochondrial genome sequence of a middle Pleistocene cave bear reconstructed from ultrashort DNA fragments. Proceedings of the National Academy of Sciences of the United States of America.

[ref-26] Dick CW, Graciolli G, Guerrero R (2016). Family streblidae. Zootaxa.

[ref-27] Dick CW, Patterson BD (2006). Bat flies: obligate ectoparasites of bats. Micromammals and macroparasites.

[ref-28] Dittmar K, Porter ML, Murray S, Whiting MF (2006). Molecular phylogenetic analysis of nycteribiid and streblid bat flies (Diptera: Brachycera, Calyptratae): implications for host associations and phylogeographic origins. Molecular Phylogenetics and Evolution.

[ref-29] Dodsworth S (2015). Genome skimming for next-generation biodiversity analysis. Trends in Plant Science.

[ref-30] Egger B, Bachmann L, Fromm B (2017). Atp8 is in the ground pattern of flatworm mitochondrial genomes. BMC Genomics.

[ref-31] Ekblom R, Galindo J (2011). Applications of next generation sequencing in molecular ecology of non-model organisms. Heredity.

[ref-32] Foster PG, De Oliveira TMP, Bergo ES, Conn JE, Sant’Ana DC, Nagaki SS, Nihei S, Lamas CE, González C, Moreira CC, Sallum MAM (2017). Phylogeny of Anophelinae using mitochondrial protein coding genes. Royal Society Open Science.

[ref-33] Goeze JAE (1782). Versuch einer Naturgeschichte der Eingeweidewürmer thierischer Körper: Mit 44 Kupfertafeln.

[ref-34] Grandjean F, Tan MH, Gan HM, Lee YP, Kawai T, Distefano RJ, Blaha M, Roles AJ, Austin CM (2017). Rapid recovery of nuclear and mitochondrial genes by genome skimming from Northern Hemisphere freshwater crayfish. Zoologica Scripta.

[ref-35] Guo A (2015). The complete mitochondrial genome of *Anoplocephala perfoliata*, the first representative for the family Anoplocephalidae. Parasites & Vectors.

[ref-36] Guo A (2016). The complete mitochondrial genome of the tapeworm *Cladotaenia vulturi* (Cestoda: Paruterinidae): gene arrangement and phylogenetic relationships with other cestodes. Parasites & Vectors.

[ref-37] Hahn C, Bachmann L, Chevreux B (2013). Reconstructing mitochondrial genomes directly from genomic next-generation sequencing reads—a baiting and iterative mapping approach. Nucleic Acids Research.

[ref-38] Hartikainen H, Bass D, Briscoe AG, Knipe H, Green AJ, Okamura B (2016). Assessing myxozoan presence and diversity using environmental DNA. International Journal for Parasitology.

[ref-39] Heintzman PD, Elias SA, Moore K, Paszkiewicz K, Barnes I (2014). Characterizing DNA preservation in degraded specimens of *Amara alpina* (Carabidae: Coleoptera). Molecular Ecology Resources.

[ref-40] Hu M, Gasser R, Chilton N, Beveridge I (2005). Genetic variation in the mitochondrial cytochrome c oxidase subunit 1 within three species of Progamotaenia (Cestoda: Anoplocephalidae) from macropodid marsupials. Parasitology.

[ref-41] Kanda K, Pflug JM, Sproul JS, Dasenko MA, Maddison DR (2015). Successful recovery of nuclear protein-coding genes from small insects in museums using Illumina sequencing. PLOS ONE.

[ref-42] Kang S, Tice AK, Spiegel FW, Silberman JD, Pánek T, Čepička I, Kostka M, Kosakyan A, Alcaˇntara DM, Roger AJ, Shadwick LL, Smirnov A, Kudryavtsev A, Lahr DJ, Brown MW (2017). Between a pod and a hard test: the deep evolution of Amoebae. Molecular Biology and Evolution.

[ref-43] Kocher A, Gantier J-C, Holota H, Jeziorski C, Coissac E, Bañuls A-L, Girod R, Gaborit P, Murienne J (2016). Complete mitochondrial genome of *Lutzomyia* (*Nyssomyia*) *umbratilis* (Diptera: Psychodidae), the main vector of *Leishmania guyanensis*. Mitochondrial DNA Part A.

[ref-44] Kocher A, Kamilari M, Lhuillier E, Coissac E, Péneau J, Chave J, Murienne J (2014). Shotgun assembly of the assassin bug *Brontostoma colossus* mitochondrial genome (Heteroptera, Reduviidae). Gene.

[ref-45] Langmead B, Salzberg SL (2012). Fast gapped-read alignment with Bowtie 2. Nature Methods.

[ref-46] Laslett D, Canbäck B (2007). ARWEN: a program to detect tRNA genes in metazoan mitochondrial nucleotide sequences. Bioinformatics.

[ref-47] Le TH, Blair D, Agatsuma T, Humair P-F, Campbell NJ, Iwagami M, Littlewood DTJ, Peacock B, Johnston DA, Bartley J, Rollinson D, Herniou E, Zarlenga S, McManus D (2000). Phylogenies inferred from mitochondrial gene orders—a cautionary tale from the parasitic flatworms. Molecular Biology and Evolution.

[ref-48] Le TH, Blair D, McManus DP (2002). Mitochondrial genomes of parasitic flatworms. Trends in Parasitology.

[ref-49] Li X, Ding S, Cameron SL, Kang Z, Wang Y, Yang D (2015). The first mitochondrial genome of the sepsid fly *Nemopoda mamaevi* Ozerov, 1997 (Diptera: Sciomyzoidea: Sepsidae), with mitochondrial genome phylogeny of *Cyclorrhapha*. PLOS ONE.

[ref-50] Li WX, Zhang D, Boyce K, Xi BW, Zou H, Wu SG, Li M, Wang GT (2017). The complete mitochondrial DNA of three monozoic tapeworms in the Caryophyllidea: a mitogenomic perspective on the phylogeny of eucestodes. Parasites & Vectors.

[ref-51] Linard B, Arribas P, Andújar C, Crampton-Platt A, Vogler A (2016). Lessons from genome skimming of arthropod-preserving ethanol. Molecular Ecology Resources.

[ref-52] Linard B, Crampton-Platt A, Gillett CP, Timmermans MJ, Vogler AP (2015). Metagenome skimming of insect specimen pools: potential for comparative genomics. Genome Biology and Evolution.

[ref-53] Littlewood D, Waeschenbach A, Nikolov P (2008). In search of mitochondrial markers for resolving the phylogeny of cyclophyllidean tapeworms (Platyhelminthes, Cestoda)—a test study with Davaineidae. Acta Parasitologica.

[ref-54] Liu Z-Q, Kuermanali N, Li Z, Chen S-J, Wang Y-Z, Tao H, Chen C-F (2017). The complete mitochondrial genome of the parasitic sheep ked *Melophagus ovinus* (Diptera: Hippoboscidae). Mitochondrial DNA Part B.

[ref-55] Lowe TM, Eddy SR (1997). tRNAscan-SE: a program for improved detection of transfer RNA genes in genomic sequence. Nucleic Acids Research.

[ref-56] Machado DJ, Janies D, Brouwer C, Grant T (2018). A new strategy to infer circularity applied to four new complete frog mitogenomes. Ecology and Evolution.

[ref-57] Machado D, Lyra M, Grant T (2016). Mitogenome assembly from genomic multiplex libraries: comparison of strategies and novel mitogenomes for five species of frogs. Molecular Ecology Resources.

[ref-58] Maddison DR, Cooper KW (2014). Species delimitation in the ground beetle subgenus *Liocosmius* (Coleoptera: Carabidae: Bembidion), including standard and next-generation sequencing of museum specimens. Zoological Journal of the Linnean Society.

[ref-59] Maldonado LL, Assis J, Araújo FMG, Salim AC, Macchiaroli N, Cucher M, Camicia F, Fox A, Rosenzvit M, Oliveira G, Kamenetzky L (2017). The *Echinococcus canadensis* (G7) genome: a key knowledge of parasitic platyhelminth human diseases. BMC Genomics.

[ref-60] Marques FP, Caira JN (2016). *Pararhinebothroides*—neither the sister-taxon of *Rhinebothroides* nor a valid genus. The Journal of Parasitology.

[ref-61] Marques FP, Reyda FB (2015). *Rhinebothrium jaimei* sp. n. (Eucestoda: Rhinebothriidea: Rhinebothriidae): a new species from neotropical freshwater stingrays (Potamotrygonidae). Folia Parasitologica.

[ref-62] Matos-Maraví P, Duarte Ritter C, Barnes CJ, Nielsen M, Olsson U, Wahlberg N, Marquina D, Sääksjärvi I, Antonelli A (2019). Biodiversity seen through the perspective of insects: 10 simple rules on methodological choices and experimental design for genomic studies. PeerJ PrePrints.

[ref-63] McCormack JE, Rodríguez-Gómez F, Tsai WL, Faircloth BC (2017). Transforming museum specimens into genomic resources. The extended specimen.

[ref-64] McCormack JE, Tsai WL, Faircloth BC (2016). Sequence capture of ultraconserved elements from bird museum specimens. Molecular Ecology Resources.

[ref-65] Nakao M, Sako Y, Ito A (2003). The mitochondrial genome of the tapeworm *Taenia solium*: a finding of the abbreviated stop codon U. Journal of Parasitology.

[ref-66] Narayanan Kutty S, Meusemann K, Bayless KM, Marinho MA, Pont AC, Zhou X, Misof B, Wiegmann BM, Yeates D, Cerretti P, Meier R, Pape T (2019). Phylogenomic analysis of Calyptratae: resolving the phylogenetic relationships within a major radiation of Diptera. Cladistics.

[ref-67] Nelson LA, Lambkin CL, Batterham P, Wallman JF, Dowton M, Whiting MF, Yeates DK, Cameron SL (2012). Beyond barcoding: a mitochondrial genomics approach to molecular phylogenetics and diagnostics of blowflies (Diptera: Calliphoridae). Gene.

[ref-68] Oliveira U, Soares-Filho BS, Paglia AP, Brescovit AD, Carvalho CJ, Silva DP, Rezende DT, Leite FSF, Batista JAN, Barbosa JPPP, Stehmann JR, Ascher JS, De Vasconcelos MF, De Marco P, Löwenberg-Neto P, Ferro VG, Santos AJ (2017). Biodiversity conservation gaps in the Brazilian protected areas. Scientific Reports.

[ref-69] Pacheco MA, Battistuzzi FU, Lentino M, Aguilar RF, Kumar S, Escalante AA (2011). Evolution of modern birds revealed by mitogenomics: timing the radiation and origin of major orders. Molecular Biology and Evolution.

[ref-70] Park J-K, Kim K-H, Kang S, Jeon H, Kim J-H, Littlewood D, Eom K (2006). Characterization of the mitochondrial genome of *Diphyllobothrium latum* (Cestoda: Pseudophyllidea)—implications for the phylogeny of eucestodes. Parasitology.

[ref-71] Petersen FT, Meier R, Kutty SN, Wiegmann BM (2007). The phylogeny and evolution of host choice in the Hippoboscoidea (Diptera) as reconstructed using four molecular markers. Molecular Phylogenetics and Evolution.

[ref-72] Pleijel F, Jondelius U, Norlinder E, Nygren A, Oxelman B, Schander C, Sundberg P, Thollesson M (2008). Phylogenies without roots? A plea for the use of vouchers in molecular phylogenetic studies. Molecular Phylogenetics Evolution.

[ref-73] Porter TM, Hajibabaei M (2018). Scaling up: a guide to high throughput genomic approaches for biodiversity analysis. Molecular Ecology.

[ref-74] Pu D-Q, Liu H-L, Gong Y-Y, Ji P-C, Li Y-J, Mou F-S, Wei S-J (2017). Mitochondrial genomes of the hoverflies *Episyrphus balteatus* and *Eupeodes corollae* (Diptera: Syrphidae), with a phylogenetic analysis of *Muscomorpha*. Scientific Reports.

[ref-75] Raposo do Amaral F, Neves LG, Resende Jr MF, Mobili F, Miyaki CY, Pellegrino KC, Biondo C (2015). Ultraconserved elements sequencing as a low-cost source of complete mitochondrial genomes and microsatellite markers in non-model amniotes. PLOS ONE.

[ref-76] Richter S, Schwarz F, Hering L, Böggemann M, Bleidorn C (2015). The utility of genome skimming for phylogenomic analyses as demonstrated for glycerid relationships (Annelida, Glyceridae). Genome Biology and Evolution.

[ref-77] Ruhnke TR, Caira JN, Cox A (2015). The cestode order Rhinebothriidea no longer family-less: a molecular phylogenetic investigation with erection of two new families and description of eight new species of *Anthocephalum*. Zootaxa.

[ref-78] Ruhnke TR, Reyda FB, Marques FPL, Caira JN, Jensen K (2017). Rhinebothriidea Healy, Caira, Jensen, Webster & Littlewood, 2009. Planetary biodiversity inventory (PBI): tapeworms from vertebrate bowels of the earth (2008–2017). Vol. 25.

[ref-79] Schattner P, Brooks AN, Lowe TM (2005). The tRNAscan-SE, snoscan and snoGPS web servers for the detection of tRNAs and snoRNAs. Nucleic Acids Research.

[ref-80] Staats M, Erkens RH, Van de Vossenberg B, Wieringa JJ, Kraaijeveld K, Stielow B, Geml J, Richardson JE, Bakker FT (2013). Genomic treasure troves: complete genome sequencing of herbarium and insect museum specimens. PLOS ONE.

[ref-81] Tan MH, Gan HM, Lee YP, Poore GC, Austin CM (2017). Digging deeper: new gene order rearrangements and distinct patterns of codons usage in mitochondrial genomes among shrimps from the Axiidea, Gebiidea and Caridea (Crustacea: Decapoda). PeerJ.

[ref-82] Tello S, Stevens RD, Dick CW (2008). Patterns of species co-occurrence and density compensation: a test for interspecific competition in bat ectoparasite infracommunities. Oikos.

[ref-83] The UniProt Consortium (2016). UniProt: the universal protein knowledgebase. Nucleic Acids Research.

[ref-84] Tilak M-K, Justy F, Debiais-Thibaud M, Botero-Castro F, Delsuc F, Douzery EJ (2015). A cost-effective straightforward protocol for shotgun Illumina libraries designed to assemble complete mitogenomes from non-model species. Conservation Genetics Resources.

[ref-85] Timmermans MJTN, Viberg C, Martin G, Hopkins K, Vogler AP (2016). Rapid assembly of taxonomically validated mitochondrial genomes from historical insect collections. Biological Journal of the Linnean Society.

[ref-86] Trevisan B, Primon JF, Marques FP (2017). Systematics and diversification of *Anindobothrium* Marques, Brooks & Lasso, 2001 (Eucestoda: Rhinebothriidea). PLOS ONE.

[ref-87] Twyford AD, Ness RW (2017). Strategies for complete plastid genome sequencing. Molecular Ecology Resources.

[ref-88] Vanhove MP, Briscoe AG, Jorissen MW, Littlewood DTJ, Huyse T (2018). The first next-generation sequencing approach to the mitochondrial phylogeny of African monogenean parasites (Platyhelminthes: Gyrodactylidae and Dactylogyridae). BMC Genomics.

[ref-89] Vellend M (2017). The biodiversity conservation paradox. American Scientist.

[ref-90] Von Nickisch-Rosenegk M, Lucius R, Loos-Frank B (1999). Contributions to the phylogeny of the Cyclophyllidea (Cestoda) inferred from mitochondrial 12S rDNA. Journal of Molecular Evolution.

[ref-91] Waeschenbach A, Webster B, Littlewood D (2012). Adding resolution to ordinal level relationships of tapeworms (Platyhelminthes: Cestoda) with large fragments of mtDNA. Molecular Phylogenetics and Evolution.

[ref-92] Wenzel RL, Tipton VJ, Kiewlicz A, Wenzel RL, Tipton VJ (1966). The streblid batflies of Panama (Diptera Calypterae: Streblidae). Ectoparasites of Panama.

[ref-93] Wickström LM, Haukisalmi V, Varis S, Hantula J, Henttonen H (2005). Molecular phylogeny and systematics of anoplocephaline cestodes in rodents and lagomorphs. Systematic Parasitology.

[ref-94] Yan D, Tang Y, Xue X, Wang M, Liu F, Fan J (2012). The complete mitochondrial genome sequence of the western flower thrips Frankliniella occidentalis (Thysanoptera: Thripidae) contains triplicate putative control regions. Gene.

[ref-95] Yang X, Liu D, Liu F, Wu J, Zou J, Xiao X, Zhao F, Zhu B (2013). HTQC: a fast quality control toolkit for Illumina sequencing data. BMC Bioinformatics.

[ref-96] Yuan S, Xia Y, Zheng Y, Zeng X (2016). Next-generation sequencing of mixed genomic DNA allows efficient assembly of rearranged mitochondrial genomes in Amolops chunganensis and Quasipaa boulengeri. PeerJ.

[ref-97] Zarowiecki M, Huyse T, Littlewood D (2007). Making the most of mitochondrial genomes–markers for phylogeny, molecular ecology and barcodes in *Schistosoma* (Platyhelminthes: Digenea). International Journal for Parasitology.

[ref-98] Zehnder M, Mariaux J (1999). Molecular systematic analysis of the order Proteocephalidea (Eucestoda) based on mitochondrial and nuclear rDNA sequences1. International Journal for Parasitology.

[ref-99] Zhang X, Duan JY, Shi YL, Jiang P, Zeng DJ, Wang ZQ, Cui J (2017). Comparative mitochondrial genomics among *Spirometra* (Cestoda: Diphyllobothriidae) and the molecular phylogeny of related tapeworms. Molecular Phylogenetics and Evolution.

[ref-100] Zhao G, Wang H, Jia Y, Zhao W, Hu X, Yu S, Liu G (2016). The complete mitochondrial genome of *Pseudanoplocephala crawfordi* and a comparison with closely related cestode species. Journal of Helminthology.

